# Descriptions of Scientific Evidence and Uncertainty of Unproven COVID-19 Therapies in US News: Content Analysis Study

**DOI:** 10.2196/51328

**Published:** 2024-08-29

**Authors:** Sara Watson, Tyler J Benning, Alessandro R Marcon, Xuan Zhu, Timothy Caulfield, Richard R Sharp, Zubin Master

**Affiliations:** 1 Biomedical Ethics Research Program Department of Quantitative Health Sciences Mayo Clinic Rochester, MN United States; 2 Department of Pediatric and Adolescent Medicine Mayo Clinic Rochester, MN United States; 3 Health Law Institute University of Alberta Edmonton, AB Canada; 4 Robert D. and Patricia E. Kern Center for the Science of Health Care Delivery Mayo Clinic Rochester, MN United States; 5 Center for Individualized Medicine Mayo Clinic Rochester, MN United States; 6 Department of Social Sciences and Health Policy Wake Forest University School of Medicine Winston-Salem, NC United States; 7 Maya Angelou Center for Health Equity Wake Forest University School of Medicine Winston-Salem, NC United States; 8 Center for Bioethics, Health and Society Wake Forest University School of Medicine Winston-Salem, NC United States; 9 Wake Forest Institute of Regenerative Medicine Wake Forest University School of Medicine Winston-Salem, NC United States

**Keywords:** COVID-19, COVID-19 drug treatment, information dissemination, health communication, uncertainty, content analysis, information sources, therapy, misinformation, communication, scientific evidence, media analysis, news report, COVID-19 therapy, treatment, public awareness, public trepidation, therapeutic, therapeutics, vaccine, vaccines, pandemic, United States, media analysis, safety, efficacy, evidence, news, report, reports

## Abstract

**Background:**

Politicization and misinformation or disinformation of unproven COVID-19 therapies have resulted in communication challenges in presenting science to the public, especially in times of heightened public trepidation and uncertainty.

**Objective:**

This study aims to examine how scientific evidence and uncertainty were portrayed in US news on 3 unproven COVID-19 therapeutics, prior to the development of proven therapeutics and vaccines.

**Methods:**

We conducted a media analysis of unproven COVID-19 therapeutics in early 2020. A total of 479 discussions of unproven COVID-19 therapeutics (hydroxychloroquine, remdesivir, and convalescent plasma) in traditional and online US news reports from January 1, 2020, to July 30, 2020, were systematically analyzed for theme, scientific evidence, evidence details and limitations, safety, efficacy, and sources of authority.

**Results:**

The majority of discussions included scientific evidence (n=322, 67%) although only 24% (n=116) of them mentioned publications. “Government” was the most frequently named source of authority for safety and efficacy claims on remdesivir (n=43, 35%) while “expert” claims were mostly mentioned for convalescent plasma (n=22, 38%). Most claims on hydroxychloroquine (n=236, 79%) were offered by a “prominent person,” of which 97% (n=230) were from former US President Trump. Despite the inclusion of scientific evidence, many claims of the safety and efficacy were made by nonexperts. Few news reports expressed scientific uncertainty in discussions of unproven COVID-19 therapeutics as limitations of evidence were infrequently included in the body of news reports (n=125, 26%) and rarely found in headlines (n=2, 2%) or lead paragraphs (n=9, 9%; *P*<.001).

**Conclusions:**

These results highlight that while scientific evidence is discussed relatively frequently in news reports, scientific uncertainty is infrequently reported and rarely found in prominent headlines and lead paragraphs.

## Introduction

The clear and accurate reporting of science to the public is imperative to maintain public trust and ensure that public health precautions are upheld [1]. Reporting science surrounding protective measures and novel unproven COVID-19 therapeutics during the first year of the pandemic was extraordinarily challenging given the hyperpoliticization of COVID-19, the misinformation or disinformation surrounding scientifically unproven and unapproved therapies, and the reliance on unsubstantiated science, for example, preprints or expert opinion [2].

Despite the number of news sources available to the public including legacy media and social media, many Americans continue to rely on traditional sources of news. Traditional media are forms of communication predating the internet and include newspapers, broadcast, and radio among others. A 2014 survey by the American Press Institute found just over 60% of Americans prefer to find news directly from news organizations compared to social media (4%), word of mouth (2%), and e-sharing with friends (1%) [3]. While social media and interpersonal communications have been implicated in the spread of misinformation and adoption of preventative behaviors, a study published in 2020 on COVID-19 information sources found traditional media sources (including television and newspapers) were the most widely used sources on COVID-19, and that the source of information individuals reported using predicted their beliefs about the virus [4].

Reviewing the content of traditional media reports is particularly important because broadcast networks, cable networks, and online and print news are viewed as the most trustworthy and reliable sources of information [4-6]. While news media has a pivotal role in communicating credible scientific evidence [7], some mainstream news outlets have propagated inaccuracies and misinformation [8,9]. Misinformation may influence health beliefs and impact compliance with public health recommendations resulting in negative health consequences [10]. The United States has the second highest prevalence of COVID-19 misinformation compared to other countries [11], which likely has impacted the health of millions of Americans [12].

The rapid release of COVID-19 research disseminated by numerous news sources with shifting health recommendations has left many feeling overwhelmed and frustrated [13,14], which may contribute to reducing trust in public health officials and institutions [15-17]. Accurate news reporting should avoid hype and speculation [18], illuminate scientific uncertainty, and portray science as iterative and evolving [19-21]. Such portrayals may serve to promote public trust and help citizens understand why public health recommendations are susceptible to change [22-24].

An examination of early COVID-19 therapeutics serves as an ideal case study on which to examine the media portrayal of scientific evidence because it was a time when there were no approved treatments, prior to the development of proven therapeutics and vaccines, and during a period of high public trepidation and scientific uncertainty regarding COVID-19 treatments. In this study, we examined how scientific evidence and uncertainty were portrayed in traditional and online US news about 3 popular and potential COVID-19 therapeutics (hydroxychloroquine, remdesivir, and convalescent plasma [CP]) when no other US Food and Drug Administration–approved treatments or vaccines were available.

## Methods

### Conceptual Framework

This research examined the portrayal of scientific evidence and uncertainty of 3 unproven COVID-19 therapeutics prior to the development of vaccines and compared its representation among the body (full text or video) of news reports with headlines and lead paragraphs. There are 2 widely understood and generally accepted paradigms of science communication that focus on the unidirectional delivery of information or active engagement with the public [25-27]. These paradigms can further be divided into 4 models of science communication, which are science literacy (also referred to as the deficit model), contextual, lay expertise, and public engagement or public participation [28]. The science literacy model is premised that information transmitted by experts aims to reduce the deficit of science knowledge among the public while the contextual model similarly transmits information, but situates information to specific audiences paying attention to culture, location, and language among other relevant contexts. Both, however, focus on the transmission of scientific information. The lay expertise model acknowledges the limitations of science and endorses “lay” knowledge as equal to scientific knowledge. The public engagement model aims to address science policy issues through active engagement and interaction with the public in a democratic process. These latter 2 models focus on improving engagement between science and society and recognize the limitations of science and refrain from giving science any more authority than other knowledge sources. Several scholars have conceptualized variations or additions to these models and outlined new goals of science communication [26,27,29,30]. However, these models remain largely theoretical and provide little practical direction toward the practice of science communication today, which involves an array of different sources (eg, internet, social media, and legacy media), actors (eg, general and science journalists, bloggers, influencers, and others), and approaches (eg, traditional journalistic norms and a plurality of practices among content contributors in the new media environment) [31]. In addition, the models provide little in terms of approaches to analyze contemporary news reports produced by traditional media outlets reported by journalists because there is no accepted conceptual framework on the public communication of science in the media and best practices to convey scientific evidence and uncertainty to the public [32,33]. Instead, our analytical approach was informed by adopting a lens of a changing science media communication landscape where public reporting of science has moved beyond conveying scientific facts and where journalists use frames to describe science accurately while maintaining audience engagement [31,34]. This, however, results in multiple challenges in accurately reporting science to the public including science hype [35]; errors of omission [36]; failure to report funding sources and conflicts of interest [37]; inadequately detailing methods, risks, and timelines [38-40]; improperly addressing scientific uncertainty; and situating science in particular contexts, for example, politics.

There is variability in the portrayal of scientific evidence and uncertainty in the news media. Some studies have shown less accurate portrayals where the results are discussed as certain and the benefits overemphasized [41-44], while other studies have demonstrated inaccuracies in reporting evidence when comparing press releases and media articles with scientific publications resulting in oversimplification, absence of quantification, lack of explicitly reporting data sources, and the absence of study limitations [45,46]. On the other hand, scientific uncertainty on climate change among other specific topics has been heightened in news media by journalists framing science as inconclusive or providing equal space for opposing viewpoints, even if one viewpoint is supported by substantial scientific consensus and evidence while the other lacks credible evidence [47-49]. News reports of scientific discoveries aiming to provide balanced perspectives may inadvertently cause a false balance and distort the public’s perception of consensus surrounding scientific evidence [50].

This research was also informed by the reality that the public often engages with snippets of information including a focus and evaluation of articles based on headlines [51,52]. Our focus on analyzing scientific evidence and uncertainty in headlines and lead paragraphs, and comparing it with the body of news reports was further informed by the primacy effect—a cognitive bias where people recall and place greater emphasis on initial pieces of information [53,54]—which may explain why headlines and lead paragraphs are especially important in shaping the audience’s understanding and judgment of news stories.

Finally, our conceptual framework was also informed by a media environment with a higher prevalence of COVID-19 health misinformation and an understanding of the efforts to correct news reports by health and scientific experts through media fact-checking and myth-busting approaches [55-58]. While several studies have shown the effectiveness of fact-checking and the correction of misinformation postexposure at altering beliefs [59-63], it remains unclear if such approaches would fully address concerns of reported health misinformation. This is partly due to differences in the focus fact-checkers and myth busters consider when aiming to correct scientific facts, the framing of science news stories by reporters, ambiguity and discordance in the interpretation of scientific facts, and that truth-telling may not always reach intended audiences [64-66]. Such approaches epistemologically rely on the assumption that facts, even in the context of scientific evidence, can always be clearly discerned from nonfactual information and that experts can identify and expose truths from falsehoods; such approaches may minimize or discount completely the complex interplay between beliefs, politics, and science [67]. Being mindful of this context, our analysis focused on how, where, and by whom scientific evidence surrounding unproven COVID-19 therapeutics was portrayed in the news stories themselves and we did not address the veracity of the scientific evidence presented in the news.

### Sampling

We chose to investigate 3 unproven products—hydroxychloroquine, remdesivir, and CP—based primarily on their popularity in US news among other factors that informed our decision. Several antivirals and immunomodulators were being considered as potential classes of therapeutics used to treat COVID-19 in the early 2020s [68]. An initial set of news database searches (discussed in the next section) identified hydroxychloroquine (4023 publications), remdesivir (2839 publications), azithromycin (416 publications), and CP (372 publications) as the most popular unproven products in US news compared to other products being considered including, lopinavir, interferon beta-1a, dexamethasone, and heparin or low molecular weight heparin. For feasibility reasons, we chose to evaluate 3 products and decided to include CP instead of azithromycin because after removing news reports discussing each product in conjunction with hydroxychloroquine, there were 344 articles discussing CP and only 57 articles discussing azithromycin. Additionally, the choice to examine hydroxychloroquine, remdesivir, and CP was supported because these products were being investigated in registered clinical studies in the United States (hydroxychloroquine, ClinicalTrials.gov NCT04714515; remdesivir, ClinicalTrials.gov NCT04257656; and CP, ClinicalTrials.gov NCT05578391) and permitted to be used to treat patients with COVID-19 by the US Food and Drug Administration (FDA; please see the following paragraphs for details).

News sources were identified using the Factiva database by the Dow Jones & Company [69]. Factiva is a commonly used research tool that provides full-text coverage of current and archival business and news information from traditional (eg, broadcast and newspaper) media and news available online. The specific news sources selected for our query were determined by popularity and coverage and were compiled based on 3 independent organizations that rank news based on circulation and internet traffic (see Table S1 in Multimedia Appendix 1) [70-72]. Having a diverse venue of sources is important since issues of unproven COVID-19 therapies have become politicized and studies have shown differing levels of depth and misinformation between news organizations.

After eliminating duplicate news sources listed by these organizations, we queried the terms hydroxychloroquine, Plaquenil, remdesivir, Veklury, and CP in the headline or lead paragraph of articles published from January 1, 2020, to July 30, 2020. The search dates were selected to ensure we captured the portrayal of scientific evidence of early unproven therapeutics to treat COVID-19 in US news. The start date of our search was chosen because a pneumonia-causing novel coronavirus was first announced on January 7, 2020, with the first confirmed US case on January 20, 2020 [73]. The end date was chosen based on clinical investigations and decisions made by the FDA on our 3 therapeutics of interest. Specifically, several clinical investigations examining the 3 potential therapeutics of interest were initiated during the first quarter of 2020, and scientific evidence about the safety and efficacy of the therapeutics was being collected and concluded [68,74,75]. Specifically, large clinical trials of hydroxychloroquine were initiated, paused, and eventually halted from late May to late June [74,76-78]. Based on clinical trial results, the FDA revoked emergency use authorization for hydroxychloroquine on June 15, 2020 [79]. Starting May 28, 2020, participants were being enrolled for a clinical trial on CP and on April 3, 2020, the FDA approved an expanded access protocol for Mayo Clinic to lead an investigation to understand the effects of CP [80]. The FDA also issued an emergency use authorization for remdesivir on May 1, 2020 [81]. Finally, the end date for our search was also chosen for practical reasons as data collection efforts began on July 31, 2020.

The search yielded 1136 reports including online and print articles, television transcripts, and videos. Application of inclusion or exclusion criteria was conducted by SW and TJB (primary coders). The coders matched the headline and news source of the web link obtained from Factiva for online media. Web-based reports with a video were considered 2 independent reports unless the video was verbatim or substantially similar to the associated article. Reports that were considered substantially similar must have had at least 1 area of similarity on the topic, main theme, concluding points, people quoted, or style (eg, debate). In instances of substantial similarity, only the most up-to-date report was included. Reports that were duplicative, unobtainable, or void of search terms were removed resulting in 1103 reports. If multiple therapeutics were discussed in a single report, the codebook was applied to each therapeutic. We randomly sampled a total of 550 news reports from our population to manage coder effort while allowing the capture of codes and themes among news reports. After random sampling, reports without substantial discussion of the COVID-19 therapeutic were excluded if they failed to satisfy codes 7-16 of the codebook (n=72). When a therapeutic was found only in an image caption, tweets, or graphics within the report, the report was also excluded (n=29). After applying exclusion criteria, a final data set of 479 individual discussions of a therapeutic was found in 449 news reports. See Figure S1 in Multimedia Appendix 1 for a flowchart providing an overview of the sampling strategy.

### Codebook

A comprehensive codebook was developed based on the literature described. The purpose of the codebook was to ensure that a consistent analytical framework was adopted by both coders and to reduce randomness in the interpretation of themes and codes. Upon developing an initial draft, the codebook was modified through the iterative analysis of 9 written articles (2 hydroxychloroquine, 2 remdesivir, and 5 CP) from different news sources with different word lengths. The draft codebook was then reviewed by a multidisciplinary team of experts in media analysis, bioethics, and health communication. Independent review of 50 reports by 2 coders (SW and TJB) showed good interrater reliability in coding for the full news report for theme and portrayal of scientific evidence. The median Cohen κ for these elements was 0.71 (IQR 0.58-0.74) with simple agreement ranging from 79% to 94%. Interrater reliability was initially poor when identifying sources of authority, with a median κ of 0.17 (IQR 0.06-0.23). Definitions were clarified to address omissions or ambiguities identified during this intercoder check. For example, evidence was defined as a formal demonstration of the effect of a treatment on the COVID-19 disease course, which includes symptoms, outcomes, side effects, or a lack thereof. In the same way, sample size or study design was added as an example of evidence details and a statement of small sample size or nonrandomized design was included in the codebook as an example of evidence limitations. Codes regarding sources of authority were modified to clarify that a subject matter expert who spoke on behalf of a government agency should not be coded as an “expert” but should be coded as a “government” source of authority. Due to poor initial reliability, modified definitions for sources of authority were formally retested by the same 2 coders on a new sample of 20 articles. Sources of authority showed good interrater reliability with a median κ of 0.73 (IQR 0.48-0.86) with simple agreement ranging from 91% to 100%. The general structure of the codebook captures metadata provided by the Factiva database (eg, word count and hyperlink), headline analysis, report analysis, scientific description, sources of authority, social context, qualitative description of video reports, and coder notes (see Multimedia Appendix 2 for final codebook with complete definitions of codes and examples).

### Analysis

The full text or video (body of news reports) of all discussions was analyzed for theme, scientific evidence, claims of safety or efficacy, and sources of authority. All terms are defined in the tables. The topical themes of news reports were identified inductively during iterative analysis of 9 articles as described above. An inductive approach permits the emergence of themes from the data and allows coders to remain open and exploratory [82]. This qualitative method is ideal for identifying themes when there is no prior knowledge about them [38,83,84]. In cases where more than 1 theme was reflected in the entire news report, the coder selected the theme that most closely resembled the news report. For text-based reports that discussed scientific evidence, details of evidence, or limitations of evidence, we also analyzed the presence of these codes within headlines and lead paragraphs. The lead paragraph was defined as the first paragraph of text with a minimum of 2 complete sentences excluding subtitles or alternate headlines just below the large print title. Coders independently analyzed approximately half the reports and jointly reviewed challenging reports and a random 10% of all reports as preplanned audits. Disagreements were resolved by consensus. Study data were recorded and managed using REDCap (Research Electronic Data Capture).

Different traits of reports (eg, discussion of evidence and discussion of specific side effects) were summarized for each therapeutic using descriptive statistics. Fisher exact tests were used to compare differences in trait frequencies. If 3×2 Fisher tests returned a *P* value of <.05, pairwise comparisons between therapeutics (ie, hydroxychloroquine - remdesivir, hydroxychloroquine-CP, and remdesivir-CP) were performed. No corrections were made for multiple comparisons. Analyses were performed in Excel (Microsoft Corp) and R (version 3.5.1; R Core Team).

### Ethical Considerations

This research study did not involve human subjects and was conducted using publicly available information; thus, ethics approval was not sought.

## Results

The data set included 479 individual discussions of hydroxychloroquine, remdesivir, and CP or a combination of any of the 3 unproven therapeutics among 449 news reports. Among 449 news reports, 191 were print, 172 were online, 52 were television transcripts, and 34 were online videos. Print news reports describe those accessed nonelectronically via PDF files from the Factiva database while online news reports and online videos were accessed by coders digitally via specific website links. News reports mostly discussed hydroxychloroquine (67%, n=299) compared to remdesivir (27%, n=122) and CP (13%, n=58). Safety or efficacy was the main theme among news on hydroxychloroquine (61%, n=182) and remdesivir (47%, n=57), whereas news reports discussing CP focused on the theme of economics, distribution, and allocation (40%, n=23). Many reports discussing hydroxychloroquine often surrounded the theme of politics (20%, n=61; Table 1).

Scientific evidence was discussed in 67% (n=322) of new discussions and was most common among discussions of hydroxychloroquine (78%, n=233) followed by remdesivir (63%, n=77) and CP (21%, n=12). All pairwise comparisons (*P*<.05; hydroxychloroquine-remdesivir *P*=.002; hydroxychloroquine-CP *P*<.001; remdesivir-CP *P*<.001; Table 2). Although scientific evidence was found in many news reports, specific publications or journals were seldom mentioned (24%, n=116). Details of scientific evidence and discussions on the limitations of evidence were found in 61% (n=198) and 26% (n=125) of news, respectively.

Among text-based news reports discussing scientific evidence, 22% (n=51) discussed scientific evidence in the headline and 51% (n=118) in the lead paragraph (Table 3). However, the details of scientific evidence were rarely found in headlines (6%, n=9) and seldom present in lead paragraphs (26%, n=39; *P*<.001). Among the 99 text-based reports that included limitations of evidence, discussions on limitations were rarely found in headlines (2%, n=2) and lead paragraphs (9%, n=9).

Very few discussions portrayed any therapeutic as safe, but 75% (n=91) of news discussions on remdesivir and 66% (n=38) of news discussions on CP were portrayed as efficacious. Only 14% (n=41) of news discussions on hydroxychloroquine portrayed the therapeutic as efficacious, and safety warnings or specific negative side effects were mainly identified in discussions on hydroxychloroquine (56%, n=168) compared to other therapeutics (Table 2). For hydroxychloroquine, most claims about safety and efficacy were offered by prominent persons (79%, n=236), almost exclusively by former US President Trump (97%, n=230). Other examples of prominent persons included Jair Bolsonaro (former president of Brazil) and Nancy Pelosi (US politician). In contrast, the government (35%, n=43) was the most frequently named source of authority for safety and efficacy claims of news on remdesivir while experts mostly made claims on CP (38%, n=22; Table 4).

**Table 1:** Thematic analysis of news reports.

	Hydroxychloroquine, n (%)	Remdesivir, n (%)	CP^a^, n (%)
Novel scientific discovery: a novel result regarding the intervention. Does not include novel results on safety or efficacy	0 (0)	0 (0)	0 (0)
Safety or efficacy: discussions about ongoing trials, study logistics, and expected results of the intervention	182 (61)	57 (47)	19 (33)
Issue of scientific integrity: misconduct by individual scientists or scientific community, including research methods, peer review, and publication or dissemination decisions	16 (5)	0 (0)	1 (2)
Misinformation: analysis of the veracity of claims or reports of fact-checking	18 (6)	2 (2)	0 (0)
Politics: reports on political figures’ claims, actions, and behaviors	61 (20)	3 (2)	0 (0)
Hope: feelings of optimism in context of society	0 (0)	2 (2)	0 (0)
Official recommendation: a statement made by a national or international government agency or governing body	3 (1)	0 (0)	0 (0)
Economics, distribution, and allocation: discussions of cost, quantity, supplies, or delivery of the intervention	12 (4)	50 (41)	23 (40)
Human interest story: individual narratives and excluding political figures	5 (2)	6 (5)	15 (26)
Other	2 (1)	2 (2)	0 (0)
Total^b^	299 (67)	122 (27)	58 (13)

^a^CP: convalescent plasma.

^b^The final data set included 479 individual discussions of therapeutics in 449 news reports.

**Table 2:** Portrayal of scientific evidence among news report discussions.

	Hydroxychloroquine, n (%)	Remdesivir, n (%)	CP^a^, n (%)	Total, n (%)
Scientific evidence^b^: a demonstration of an effect on the disease course or resultant side effects from the intervention.	233 (78)	77 (63)	12 (21)	322 (67)
Details of scientific evidence^c^: specific details about the design or methodology of evidence.	152 (65)	40 (52)	6 (50)	198 (61)
Limitations of evidence: aspects of evidence or a study presented as shortcomings.	104 (35)	12 (10)	9 (16)	125 (26)
Publication or journal^b^: identifying a specific publication or journal named.	102 (34)	11 (9)	3 (5)	116 (24)
Portrayed as safe: portrayed therapeutic as safe using keywords, for example, safe or discussing minimal risks or side effects.	41 (14)	6 (5)	8 (14)	55 (11)
Portrayed as efficacious^b^: portrayed therapeutic as efficacious by using keywords, for example, effective and helpful.	41 (14)	91 (75)	38 (66)	170 (35)
Safety warnings or side effects^b^: discusses specific health or safety risks, for example, death.	168 (56)	1 (0.8)	1 (2)	170 (35)

^a^CP: convalescent plasma.

^b^Statistically significant result for 3×2 Fisher exact test; *P*<.001.

^c^Evidence details were analyzed only if the report included evidence; n=322.

**Table 3:** Scientific evidence, details, and limitations in headline or lead paragraphs^a^.

	Hydroxychloroquine, n (%)	Remdesivir, n (%)	CP^b^, n (%)	Total, n (%)
Scientific evidence in headline^c^, n (%)	42 (24)	8 (13)	5 (42)	51 (22)
Scientific evidence in lead paragraph^c^, n (%)	92 (53)	26 (43)	8 (67)	118 (51)
Total reports with scientific evidence in headline or lead paragraph, n	173	61	12	233
Details of scientific evidence in headline^c^, n (%)	8 (7)	1 (3)	0 (0)	9 (6)
Details of scientific evidence in lead paragraph^c^, n (%)	28 (25)	10 (30)	1 (25)	39 (26)
Total reports with details of scientific evidence in headline or lead paragraph, n	113	33	4	150
Limitations of evidence in headline^c^, n (%)	2 (3)	0 (0)	0 (0)	2 (2)
Limitations of evidence in lead paragraph^c^, n (%)	5 (6)	3 (25)	1 (11)	9 (9)
Total reports with limitations of evidence in headline or lead paragraph, n	78	12	9	99

^a^The following definitions were used. Scientific evidence: a demonstration of an effect on the disease course or resultant side effects from the intervention. Details of scientific evidence: specific details about the design or methodology of evidence. Limitations of evidence: aspects of evidence or a study presented as shortcomings.

^b^CP: convalescent plasma.

^c^A single news report was included in the analysis of more than 1 therapeutic when the report discussed multiple therapeutics.

**Table 4:** Sources of authority among news report discussions.

	Hydroxychloroquine, n (%)	Remdesivir, n (%)	CP^a^, n (%)	Total, n (%)
Prominent person^b^: a nonmedical, nonscientist person such as celebrities and politicians	236 (79)	9 (7)	1 (2)	246 (51)
Institution: an academic institution, medical center, or hospital	22 (7)	9 (7)	9 (16)	40 (8)
Expert^b^: a trained professional such as clinician, scientist, or clinician-researcher speaking independently of an institutional affiliation	146 (49)	31 (25)	22 (38)	199 (41)
Government^b^: a federal or state government organization such as the Centers for Disease Control and Prevention, Food and Drug Administration and World Health Organization	180 (60)	43 (35)	12 (21)	235 (49)

^a^CP: convalescent plasma.

^b^Statistically significant result for 3×2 Fisher exact test; *P*<.001.

## Discussion

### Principal Findings

As reports spread of a novel coronavirus causing unprecedented hospitalizations and mortality, both medical specialists and everyday persons anxiously scrambled to learn as much and as fast as possible about prevention and potential treatments. Our analysis revealed that much coverage of the off-label use of hydroxychloroquine centered on politics and the safety and efficacy of the product. The politics theme dominated discourse surrounding hydroxychloroquine due to its endorsement by former President Trump as seen in other news media analyses [58,85]. During the months of March and April 2020, former President Donald Trump put forth hydroxychloroquine as a “game changer” after a study suggested efficacy in vitro. Propagation of statements surrounding the safety and efficacy of hydroxychloroquine had far-reaching effects, including increased sales by hospitals and the death of persons who took a similar product as prophylaxis [86]. While safety or efficacy discussions were also found in remdesivir and CP, limited knowledge of its antiviral properties may have played a significant role in shaping public discourse on the potential safety and efficacy of the drug. Conversations about CP instead focused on the human interest theme explicating the need for plasma donors and inspiring potential COVID-19 survivors to donate.

The results showed substantial heterogeneity in the sources of authority asserting claims of safety or efficacy of the COVID-19 therapeutics among the 449 reports. On many occasions, scientific claims of the safety and efficacy of a therapeutic were portrayed by prominent persons and were compared to the evidence discussed by experts. Contrary opinions on evidence of hydroxychloroquine delivered by politicians and experts may have inadvertently contributed to a false sense of scientific disagreement as news reports most frequently reported on claims made by a single prominent person but also mentioned the lack of scientific evidence supporting such claims. Presenting directly opposing viewpoints as equal when evidence or a scientific consensus is actually weighted is referred to as a false balance that can cause public uncertainty [87]. False-balance has been shown to distort public perceptions of consensus among experts resulting in people identifying greater disagreement and controversy than what is actually present [50]. Making such comparisons is problematic because nonscientific experts may interpret data toward a political advantage and their influence could have troubling downstream consequences when disseminated, for example, physicians prescribing unproven products and reduced intentions to adopt health behaviors [88-90]. Though the majority of news reports on hydroxychloroquine published the safety or efficacy claims made by prominent persons, very few reports actually portrayed hydroxychloroquine as efficacious, and safety warnings or specific side effects were frequently included.

While we found scientific evidence of therapeutics was portrayed in the body of news reports, mostly on hydroxychloroquine, limitations of evidence outlining scientific uncertainty were seldom seen, and rarely found in prominent locations (headlines and lead paragraphs) of news reports. With only about 40% of the public delving past headlines or lead paragraphs when reading articles [3,91], it is unlikely readers would grasp the scientific uncertainty associated with an unproven therapeutic. The dissemination of evolving science without expressing scientific uncertainty and the knowledge gap created by the iterative scientific process may have indirectly resulted in public confusion and minimized how large of a threat individuals believed the virus posed [92,93]. This confusion and doubt permitted some actors to promote disinformation surrounding the safety and efficacy of unproven therapeutics [8,21]. Journalists may be reluctant to express uncertainty of evidence in their reports out of fear their audience may react negatively toward such ambiguity [94] or the journalist may not be equipped to address a study’s findings, especially its limitations. Scientists on the other hand are reportedly reluctant to express uncertainty fearing reporters may lose interest and that the public might misinterpret the science or doubt scientists [95,96]. However, empirical studies have shown that neither conveying uncertainty nor presenting scientific limitations affect a person’s understanding of a topic, beliefs, or trust in science [97-99]. This data, along with our findings, suggest that uncertainty should be explained more frequently in news reports and placed in headlines and lead paragraphs to adequately support the public’s understanding of scientific evidence and uncertainty [30,87]. Further research comparing scientific evidence and expressions of uncertainty between the body of news reports versus headlines and lead paragraphs on different scientific topics is needed. Research examining key factors influencing journalists’ and news editors’ decision-making process regarding news headlines and lead paragraphs is important to inform updated journalistic guidelines and editorial policies aimed at reducing false balance. Research collaboration between journalists, scientists, and communication researchers to identify effective communication strategies and journalistic practices to ensure accurate and nuanced representation of scientific information in news reporting would be valuable.

The accurate reporting of scientific evidence and uncertainty is the collective responsibility of multiple professionals including reporters, editors, public health officials, and medical and scientific experts. While evidence from experts may be based on scientific facts, expert correspondents should make clear scientific uncertainty, avoid hyperbolic statements, and explain how the state of knowledge may change [22]. Medical doctors and scientists should not express unjustified certainty when forecasting the safety and efficacy of unproven therapeutics [100]. Despite the lack of consensus, reporters should offer greater context or altogether avoid framing unsubstantiated claims by prominent people alongside evidence-based claims from experts as it may provide a false impression of genuine scientific uncertainty. Editors should refrain from sensationalizing headlines or framing lead paragraphs that do not reflect evidence or study limitations. Instead, journalists and experts alike should focus on the accurate portrayal of the scientific studies presented in the report and aim to provide an accurate reflection of the safety and efficacy of unproven products to help the public understand why there is incomplete evidence and a lack of definitive recommendations. Prioritizing the accurate portrayal of science may help to rebuild public trust in experts, minimize risky behaviors, and ensure compliance with public health recommendations.

### Limitations

Our study has several limitations worth mentioning. First, the analysis included only news sources within the United States, all of which were written in English and thus may not cater to news outside of the country and written in different languages. Second, the analysis focused on traditional, mainstream news sources and we excluded social media. Although much COVID-19 information and misinformation were disseminated through social media contributing to an infodemic, traditional news remains the major form of information transfer, especially among older Americans, which is why we focused on evaluating traditional news sources [101]. Third, we chose to analyze approximately half the news reports in our population and the sample may not be representative of the entire news discourse within the full data set. Fourth, the data collection and analysis was restricted to the 3 most popular unproven COVID-19 therapeutics and other therapeutics during that period may be portrayed differently in US news. Finally, the results were not stratified based on different political leaning of news sources included in our analysis, which could affect how scientific evidence and uncertainty were portrayed in our data set.

### Conclusions

News media plays a significant role in informing the public by serving as a crucial link between public health authorities interpreting scientific evidence and determining its implications and the public. Rapid publication of peer-reviewed and nonreviewed science allowed the media to bring breaking news about discoveries of COVID-19 therapeutics to the public and convey public health recommendations [18]. Prioritizing the accurate portrayal of science can help shape the impact of a public health emergency by influencing public perceptions and activities such as minimizing risky behaviors and encouraging compliance with public health recommendations. Publicizing that the news media source is likely to share frequent updates from scientific findings and communicating information as the best-known information at the time may help build public trust in scientific experts and decrease feelings of doubt and anxiety. The accurate reporting of scientific evidence and uncertainty is the collective responsibility of journalists and science communicators. Public trust in science can be strengthened by acknowledging evidentiary limitations, avoiding a false sense of disagreement, and portraying science as an iterative, self-corrective process that generates reliable knowledge.

## Appendix

Multimedia Appendix 1 Methods.

Multimedia Appendix 2 Codebook.

## Abbreviations

CP: convalescent plasma
FDA: US Food and Drug Administration
REDCap: Research Electronic Data Capture
