# Supplementary material for: Descriptions of Scientific Evidence and Uncertainty of Unproven COVID-19 Therapies in US News: Content Analysis Study
Source: JMIR Infodemiology. 2024 Aug 29;4:e51328. doi: 10.2196/51328 (PMC11393509; doi:10.2196/51328)
Supplement: Multimedia Appendix 1 [file infodemiology_v4i1e51328_app1.docx]

**Multimedia Appendix 1****: Methods**

To build our data set of news sources, we included all 12 news sources provided by statista.com (https://www.statista.com/statistics/184682/us-daily-newspapers-by-circulation/), the top 15 of 100 news websites listed in the Feedspot blog (https://blog.feedspot.com/usa_news_websites/) were selected, and all 10 newspaper sources listed by Agility PR solutions (https://www.agilitypr.com/resources/top-media-outlets/top-10-daily-american-newspapers/). After removing duplicate news sources among the three lists (Statista, Feedspot, and Agility PR) , 23 news sources were included our data collection method as shown in Table 1.

Table S1: News sources (n=23) included from Factiva database search

| The New York Times - All sources | HuffPost - All sources | Fox News - All sources | USA Today - All sources |
| --- | --- | --- | --- |
| Reuters - All sources | Politico | Yahoo - All sources | NPR: News Special; NPR: Morning Edition; NPR: Weekend Edition - Saturday; NPR: Weekend Edition - Sunday |
| Los Angeles Times – All sources | Breitbart News Network | New York Post | NBC Network - All sources |
| ABC News: 20/20; ABC News: Good Morning America; ABC News: Nightline; ABC News: Special Report; ABC News: World News Saturday; ABC News: World News Sunday; ABC News | CBS Network - All sources | CNN - All sources | The Wall Street Journal - All sources |
| Washington Post - All sources | Star-Tribune (Minneapolis-St. Paul) | Newsday (N.Y.) | Chicago Tribune - All sources |
| The Boston Globe - All sources | New York Daily News | Tampa Bay Times (Fla.) |  |

The articles collected from the Factiva database search were screened to exclude duplicates or substantively similar articles, to ensure the article was focused on a Covid-19 therapeutic of interest and was accessible via web browser. A second application of exclusion criteria was applied to a simple random sample. Figure 1 displays the application of inclusion and exclusion criteria to the sample of new reports used in this analysis


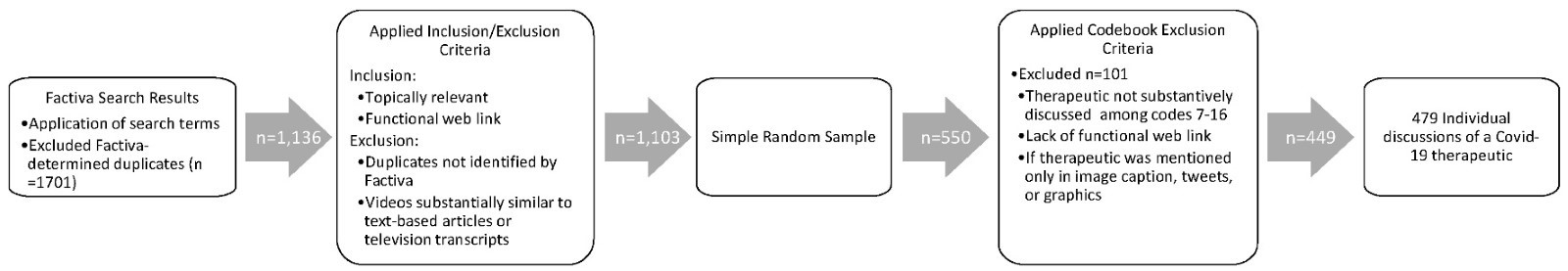
Figure S1: Inclusion and Exclusion Criteria Flowchart
