# Supplementary material for: Descriptions of Scientific Evidence and Uncertainty of Unproven COVID-19 Therapies in US News: Content Analysis Study
Source: JMIR Infodemiology. 2024 Aug 29;4:e51328. doi: 10.2196/51328 (PMC11393509; doi:10.2196/51328)
Supplement: Multimedia Appendix 2 [file infodemiology_v4i1e51328_app2.docx]

**Multimedia Appendix 2: Codebook**

#### Our codebook was applied to all body and headline/lead paragraph analysis as indicated.

#### Factiva Meta-data

1. Title
2. Internet Hyperlink
3. Date
4. Word Count

#### Report Overview

1. Which of the following COVID-19 interventions are named in the report? Select all that apply.
2. Convalescent Plasma
3. Hydroxychloroquine (specific product or brand name)
4. Remdesivir (Specific product or brand name)
5. Other, write in
6. What is the single main theme of the report (body text)?
7. Novel scientific discovery/result
8. *A novel result regarding the intervention. Does not include novel results on safety or efficacy.* Safety and/or efficacy of the product
9. *Discussions about ongoing trials, study logistics, and expected results of the intervention.* Issue of scientific integrity
10. *Misconduct by individual scientists or scientific community, including research methods, peer review, publication/ dissemination decisions* Misinformation

*Analysis of the veracity of claims or reports of fact-checking.. Fact-checking of a political figure is Misinformation, whereas simply reporting on a political figures’ claims is Politics.*

1. Politics

*Reports on political figures’ claims, actions, and behaviors.* e.g., *touting of hydroxychloroquine by current administration*

1. Hope

*Feelings of optimism in context of society. Hopefulness or optimism for individual recovery is Human interest story.*

1. Official recommendation by authoritative person/body

*A statement made by a national or international government agency or governing body*

1. Economic or distribution or allocation (scarcity) of the product

*Discussions of cost, quantity, supplies, or delivery of the intervention.*

1. Human interest story

*Individual narratives, excluding political figures. Report about a political figure (elected official, civil servant) is Politics.*

1. Other, Write Description.
2. What was the tone towards the intervention as a treatment for COVID-19?
3. Positive
4. Neutral
5. Negative

#### Scientific Description: repeat for each intervention named in a report

1. Does the report discuss the evidence for treating COVID-19 and its symptoms?

*Evidence includes any formal demonstration of the effect of a treatment on the disease course of COVID-19 or on the resultant side effects of a treatment. May include change in symptoms, outcomes, or lack thereof. May also include an expert’s summary of the evidence. (Expert may be specific individual or group.)*

- 1. No
  2. Yes

1. If yes in 7., were specific scientific/clinical studies discussed?

*Randomized controlled trials, clinical trials, chart analyses, observational, cohort size etc. Must include further details beyond the simple existence of the study- for example, methodological details such as sample size or randomization. Study must be about the intervention for the treatment/prevention of COVID-19.*

1. Does the report discuss the methodological shortcomings (e.g., small sample, non-randomized, retrospective) of evidence for treating COVID-19 and related symptoms?

*Includes discussing the limitations of at least one study or discussing the relative limitations of one study compared to another.*

- 1. No
  2. Yes

1. Was the intervention portrayed as safe?

*Must have safety language e.g., mention of little risk, side effects, adverse events or use of the word “safe.”*

- 1. No
  2. Yes

1. Was uncertainty about the intervention’s safety portrayed?

*e.g., may only be effective for certain populations or product has side-effects. Statements questioning the overall safety profile or stating that the product’s safety is unknown is portrayed uncertainty. If a treatment is portrayed as unambiguously unsafe, this alone would not be a portrayal of uncertainty. Qualifying or hedging language about specific side effects (e.g., may cause heart palpitations) is not portrayed uncertainty.*

- 1. No
  2. Yes, description

1. Were specific warnings or side effects of the intervention or combination of interventions in the report?

*e.g., risk of death*

- 1. No
  2. Yes, description

1. Was the need for or a call for additional testing or evidence to demonstrate safety explicitly stated in the report?
   1. No
   2. Yes
2. Was the intervention portrayed as efficacious?
   1. No
   2. Yes
3. Was uncertainty about the efficacy of the intervention portrayed?

*e.g., ineffective, experimental, unproven, promising. The uncertainty must be portrayed in a single statement and not derived from a global assessment of competing statements in the report. If a treatment is portrayed as unambiguously ineffective, this alone would not be a portrayal of uncertainty. The use of qualifying or hedging language such as “may,” “some,” “could” is not portrayed uncertainty.*

- 1. No
  2. Yes, description

1. Was the need for or call for additional testing or evidence to demonstrate efficacy explicitly stated in the report?

*e.g., ongoing studies including plans to complete a study or trial, future studies, or forthcoming data collection or dissemination of data or new information not currently available or present in the report is discussed in the report.*

- 1. No
  2. Yes

#### Sources of Authority: repeat for each source of authority named in the report.

*The claim does not need to be specific but rather can be inferred from an action e.g.,. taking a drug, stopping a trial, approving a drug, revoking/stopping use (Neither starting a trial nor distributing an intervention are not actions that infers efficacy or safety). Reporting results of a clinical or scientific study is considered a claim about the safety/efficacy of the intervention. The source of authority should be speaking about the intervention and COVID-19. All quotes have to be active quotes meaning they cannot be repurposed from another article/source.*

1. Did a specific prominent person verbally claim or otherwise demonstrate or suggest that the intervention was either (in)efficacious and/or (un)safe?

*A prominent person is a non-medical, non-scientific person e.g., celebrities, politicians, actors. TV show hosts and reporters are not considered prominent persons.*

- 1. No
  2. Yes, description of person

1. If yes 17., was the prominent person quoted?
   1. No
   2. Yes
2. Did a specific expert verbally claim or otherwise demonstrate or suggest that the intervention was either (in)efficacious and/or (un)safe?

*An expert is a credentialed person in the medical or scientific field e.g., scientist, clinician, M.D., D.O., Ph.D.. If an expert is explicitly portrayed as speaking on behalf of an institution or academic medical center, the source of authority for the claim is not an expert but rather an institution, 23.*

- 1. No
  2. Yes, description of expert

1. If yes 19., was the expert quoted?
   1. No
   2. Yes
2. Did a specific government agency or governing body verbally claim or otherwise demonstrate or suggest that the intervention was either (in)efficacious and/or (un)safe?

*e.g., CDC, NHS, WHO, includes a government employee named in a report with the name of the government or governing body employer.*

- 1. No
  2. Yes, description of the government agency or governing body

1. If yes 21., was the government agency or governing body quoted?
   1. No
   2. Yes
2. Did a specific academic institution or hospital verbally claim or otherwise demonstrate or suggest that the intervention was either (in)efficacious and/or (un)safe?

*If an academic or institutional affiliation is mentioned for an expert and the expert is portrayed as speaking independently, the source of authority is not institution but rather an expert, 19.*

- 1. No
  2. Yes, description of academic institution or hospital

1. If yes 23., was the academic institution or hospital quoted?
   1. No
   2. Yes
2. Was a specific scientific publication named?

*e.g., journal names, publication titles, mention of scientific publication.*

- 1. No
  2. Yes

1. If yes 25. What was the tone of the scientific publication towards the intervention as a treatment for COVID-19?
2. Predominantly optimistic
3. Predominantly pessimistic
4. Neither optimistic nor pessimistic

#### Social Context

1. Was the cost of the intervention discussed?
   1. No
   2. Yes
2. Was the quantity, availability, rationing or stockpiling of the intervention discussed?
   1. No
   2. Yes

#### Qualitative Descriptions of Video Reports

1. Provide a general description of the video in terms of what it is about in terms of content, any general aspects about who is speaking, was their animation, special effects etc.
2. List video production units or owners. List links to external sources or references

#### Coder Notes

1. Select for Coder Audit
2. Yes
3. Exclude Report
4. Yes
5. If yes in 32., is the report excluded because of no substantive discussion (#7-16 = 0)
6. Comments
